# Periphytic biofilms-mediated microbial interactions and their impact on the nitrogen cycle in rice paddies

**DOI:** 10.1016/j.eehl.2022.09.004

**Published:** 2022-11-05

**Authors:** Zhihao Chen, Jan Dolfing, Shunyao Zhuang, Yonghong Wu

**Affiliations:** a State Key Laboratory of Soil and Sustainable Agriculture, Institute of Soil Science, Chinese Academy of Sciences, Nanjing 210008, China; b Zigui Three Gorges Reservoir Ecosystem, Observation and Research Station of Ministry of Water Resources of the People's Republic of China, Yichang 443605, China; c University of Chinese Academy of Sciences, Beijing 100049, China; d Faculty of Energy and Environment, Northumbria University, Newcastle Upon Tyne NE1 8QH, UK

**Keywords:** Periphytic biofilms, Microbial aggregates, Regulation, Nitrogen cycle

## Abstract

Rice paddies are unique waterlogged wetlands artificially constructed for agricultural production. Periphytic biofilms (PBs) at the soil–water interface play an important role in rice paddies characterized by high nutrient input but low utilization efficiency. PBs are composed of microbial aggregates, including a wide variety of microorganisms (algae, bacteria, fungi, protozoa, and metazoa), extracellular polymeric substances and minerals (iron, aluminum, and calcium), which form an integrated food web and energy flux within a relatively stable micro-ecosystem. PBs are crucial to regulate and streamline the nitrogen cycle by neutralizing nitrogen losses and improving rice production since PBs can serve as both a sink by capturing surplus nitrogen and a source by slowly re-releasing this nitrogen for reutilization. Here the ecological advantages of PBs in regulating the nitrogen cycle in rice paddies are illustrated. We summarize the key functional importance of PBs, including the intricate and delicate community structure, microbial interactions among individual phylotypes, a wide diversity of self-produced organics, the active adaptation of PBs to constantly changing environments, and the intricate mechanisms by which PBs regulate the nitrogen cycle. We also identify the future challenges of microbial interspecific cooperation in PBs and their quantitative contributions to agricultural sustainability, optimizing nitrogen utilization and crop yields in rice paddies.

## Highlights

• Periphytic biofilms (PBs) are active microbial aggregates ubiquitously distributed in wetlands.
• PBs form a stable and intricate food web catalyzing biogeochemical cycling of soil nitrogen.
• PBs in rice paddies can improve nitrogen utilization efficiency by plants.
• PBs-mediated microbial interactions facilitate the nitrogen cycle cooperation among microorganisms, while self-produced organics and genomic adaptation are beneficial for the nitrogen cycle regulation by PBs.

## Graphical abstract

**Figure:**
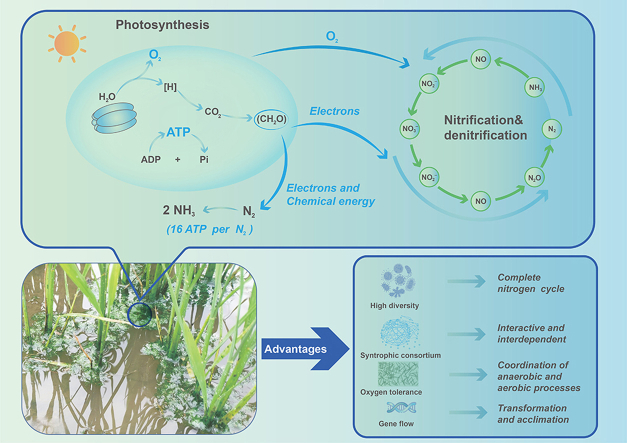

## 1 Introduction

Rice feeds over 50% of the world population [1], and is central to global food security and environmental sustainability [2]. Growing rice requires nitrogen, and synthetic fertilizers are heavily used to achieve higher yields [3]. However, the nitrogen utilization rate of chemical fertilizers is only 35%–50% in rice paddies [4,5]. Excessive use of nutrients has turned rice paddies into sources of non-point pollution [6,7]. The loss of reactive nutrients causes eutrophication and the degradation of soils, which negatively affects plant productivity [8]. Therefore, proper practices are needed to improve nitrogen utilization efficiency, reduce the harmful effects of synthetic fertilizers on soil health, and ensure sustainable rice productions.

Periphytic biofilms (PBs) emerged as a key component of the rice ecosystem because abundant nutrient supply and suitable light at the soil-water interface in rice paddies provide an ideal environment for the formation and growth of PBs [9]. PBs are microbial aggregates with intertwined abiotic substances, growing on flooded substrates, also known as natural biofilms, that are widely distributed in aquatic ecosystems [10]. PBs contain a variety of organisms, have a complex biotic composition [11] and develop a complete food web [12]. Benefiting from the complex and diverse communities in microbial aggregates, intricate interactions between species are fostered, promoting steady and functional PBs [13]. The rich genetic diversity in and compact spatial distribution of PBs provide for flexible gene flow, contributing to rapid transformation and adaptation in response to environmental stresses [14].

As for the disconnect between excessive nitrogen fertilizers and low utilization rate, PBs entrap and accumulate surplus nitrogen to subsequently release nitrogen into the soil and overlying water when its biomass is decomposed [15,16], thus acting as a nutrient sink and buffer, supporting rice growth and reducing pollution [17]. Underpinning such nitrogen cycle regulation impacts, the core steps include nitrogen fixation, nitrogen removal mainly through ammonia volatilization and denitrification, as well as other interlaced nitrogen-transforming reactions. Although each reaction is accomplished by a single or a few species [18,19], the functional microorganisms are actually interacting, forming a nitrogen cycle network [20]. The variable environment in rice paddies does not meet all the physiological needs of one microorganism [21]. Accordingly, PBs with metabolic diversity and symbiotic relationships possess built-in superiority in running an efficient nitrogen cycle regulation, especially the capabilities to fine-tune N utilization and withstand the stress of oxygen, supply sufficient ATP and electron donors for nitrogen transformation, and adapt rapidly at the gene level.

Despite their vast potential in harnessing the nitrogen cycle, strengthening nitrogen utilization, and reducing non-point source pollution, PBs have not received much attention, and few comprehensive reports on their distinctive features and functions with respect to nitrogen behavior have been conducted. Thus, this review aims to (a) clarify the characteristics of PBs, (b) elucidate the mechanism and significance of PBs on the nitrogen cycle in rice paddies, and (c) identify the challenges and prospects of PBs and PBs management for the future.

## 2 Unique structure of PBs

**Fig. 1:**
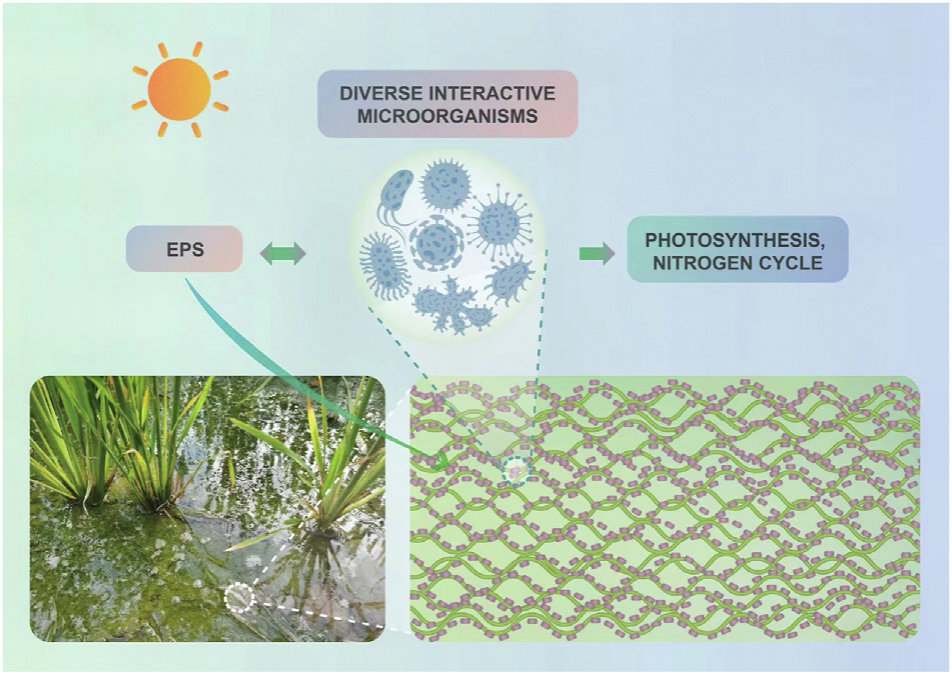
The biotic composition of PBs in rice paddies. Bacteria, algae, fungi, protozoa and metazoa secrete extracellular polymeric substances (EPS), and in return EPS intertwine the microorganisms within PBs, shaping the reticulated spatial structure of PBs. This biotic composition of PBs is conducive to promoting syntrophic interactions of diverse microorganisms and maintaining various activities such as photosynthesis and the nitrogen cycle processes.

PBs are microbial aggregates with taxonomic diversity, and are composed of metal oxides (iron, manganese, and aluminum oxides), organics, small amounts of minerals, and microorganisms with a wide range of biological types, such as manifold species (algae, bacteria, fungi, protozoa, and metazoa), organized in intricate food webs. It is this very composition of PBs that forms their distinct ecological functions (Fig. 1).

### 2.1 Abiotic matrix

The metal oxides of PBs mainly encompass iron, manganese, and aluminum oxides, which in turn contribute to a distinct enrichment of trace metals [22]. In the paddy ecosystem, the metal oxides provide a relatively stable environment for microorganisms, serving as a growth matrix of PBs [23]. Minerals such as iron and aluminum compounds make trace elements available for cells and promote intercellular interactions. Microorganisms can interact with minerals in various ways, affecting the formation and function of PBs, and ultimately have an important impact on the biogeochemical processes of many elements in their environment, such as nitrogen, iron, sulfur, etc. [23,24].

### 2.2 Biotic composition

PBs are dominated by a diversity of microorganisms encapsulated in an abiotic matrix [25]. Bacteria and algae are the core microorganisms of PBs, and the extracellular polymeric substances (EPS) they produce further shape the PBs structure [26] and their functional properties [27]. Fungi can use a wide range of organics, especially multi-carbon and even refractory organics [28]. Algae and autotrophs dominate PBs exposed to sunlight, which usually appear on the rocks of open canals and streams [10]. Protozoa in mature PBs constantly prey on bacteria, playing an active role in keeping the bacteria in an active physical state. Metazoa are multicellular animals such as rotifers, nematodes, ciliates, insects, and their larvae [10].

EPS are a mixture of organic polymers secreted by algae and bacteria, and other microorganisms, mainly composed of proteins, polysaccharides, humic acids, nucleic acids, and lipids [26], which are vital for the formation, structure and function of PBs [29]. EPS can be subdivided into bound extracellular polymeric substances (BEPS, including sheath, capsule polymer, gel, loosely bound polymer and attached organic materials) and soluble extracellular polymeric substances (SEPS, including soluble macromolecules, colloids and mucus) [30]. EPS affect the physical and chemical properties of PBs by affecting mass transfer, surface charge, flocculation, sedimentation, dehydration, adhesion and the formation of biofilms [31].

## 3 Characteristics of PBs as microbial aggregates

It is an intrinsic characteristic of PBs to exist in the form of microbial aggregates. Exploiting this structure, PBs form a stable and intricate food web, where microorganisms are interdependent and functionally complementary, allowing for effective communication and transformation to resist, acclimate and adapt in response to environmental fluctuations.

### 3.1 Microbial aggregates and complex food web

Fundamentally different from the physiological function of microorganisms in pure cultures, PBs exist in the form of complex, diverse, and highly dynamic communities. High biodiversity promotes intricate interactions between diverse species and allows for a steady microbial community with stable natural functions [13]. Densities of microbiota (including bacteria, algae, fungi, protozoa, metazoa and viruses) within such aggregates well exceed those of planktonic systems [32].

Typically, bacterial groups within PBs in rice paddies are dominated by *Proteobacteria* and cyanobacteria [33,34]. Normally, there are abundant cyanobacteria, Bacillariophyta, and Chlorophyta within PBs in rice paddies as a result of natural sunlight, thus providing carbon sources for heterotrophic microorganisms [35]. Fungi within PBs accelerate the decomposition of organics, while protozoa, metazoa, and viruses control the growth of the microbial aggregates and alter their diversity, structure and function [36]. Specifically, algae and bacteria in PBs can be prey for micro grazers, including protozoa and metazoa, contributing to the formation of an interlaced trophic hierarchy, essentially a food web within the aggregates [37]. Bacteria and microalgae may run a high predation risk from micro grazers by coexisting with them, but prey species do not submit to predators categorically: bacteria and algae are in fact largely resistant to microbial grazing [38]. Consequently, most microorganisms in PBs are organized congruently in space and integrated in metabolism, forming a dynamic homeostasis that allows them to survive under environmental stress [39] and provides unique ecological functions. Such food web-based interactions possibly alter the material and energy fluxes significantly [32], and then maintain a relatively independent, self-contained, and stable ecosystem.

### 3.2 Aggregates and syntrophy

**Fig. 2:**
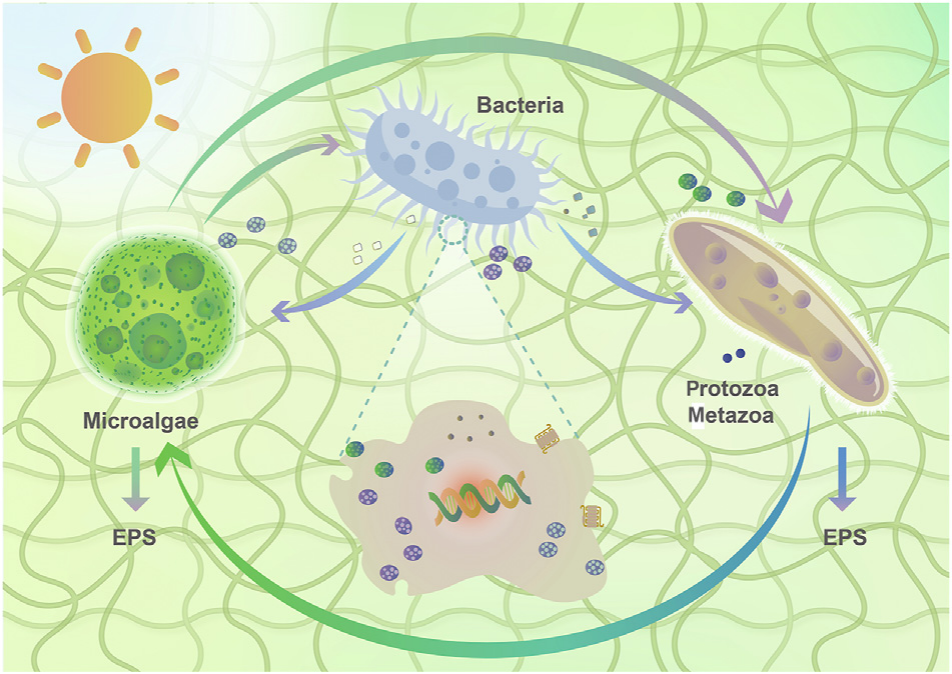
Syntrophic interactions shape the microbial communities of PBs and their capability of nutrient cycling. Syntrophic interactions within PBs occur on the self-organized reticulated structure. Microalgae provide oxygen and organics for bacteria and microfauna, and obtain carbon dioxide and minerals in return. Bacteria are the driving force to complete nutrient cycling, but they will also be preyed on by microfauna. Benefiting from such syntrophic interactions, nutrient transformation by PBs, including the nitrogen cycle reactions, is efficient.

Symbiosis and especially mutualism, where both partners benefit from living together, is ubiquitous throughout the biosphere. As for PBs, their complex composition offers ample opportunities for such beneficial interactions; as biofilm microorganisms can survive attached to substrates and to each other, generally surrounded by and embedded in EPS [40], assembling the microorganisms in a distinct reticulated structure [37]. Symbiotic relationships between microalgae and bacteria in PBs are relatively common. The importance of algae–bacteria interactions has been well recognized: classified as primary producers and decomposers they serve as the foundation of the aggregates. Microalgae provide organics (e.g., carbohydrates, proteins) as well as a habitat [41] for bacteria growth, while bacteria in turn consume the oxygen produced by the microalgae, decreasing the photosynthetic oxygen tension and releasing carbon dioxide used by the microalgae [42]. These microorganisms may establish additional cooperative interactions through the exchange of metabolites, resulting in an overall increase in biomass productivity and nutrient removal efficiency [43] (Fig. 2). Another type of syntrophic interaction is found in methanogenic communities where thermodynamics dictates the concentration limits of the metabolic substrates and products of the organisms involved [44].

Competition and antagonism also occur in PBs [45]. Specific chemicals excreted by algae can weaken bacterial quorum sensing (QS) [46]. Similarly, bacteria can secrete metabolites with algicidal characteristics [47]. Mutualism and antagonism often co-occur in PBs, it is the very cooperation and competition between microorganisms that drive PBs to metabolize more efficiently.

### 3.3 Aggregates and interspecific communication

A microbial aggregate is a society or micro-world by and of itself, where the residents communicate and contact each other. One type of intercellular communication mechanism is QS, the exchange of small signaling molecules between cells, such as N-acyl-homoserine lactones (AHLs), autoinducers (AI-2) and autoinducing peptides (AIP), which participate in coordinating gene expression and regulating physiological behavior [48].

Communication between bacteria via QS has been widely studied, especially AHLs-based QS in Gram-negative bacteria. Spatial distance is a limiting factor in cell communication, yet the high microbial diversity and long-term closeness of various microorganisms within microbial aggregates allow for frequent interactions through QS [49]. Indeed, the concentration of AHLs can be up to 1000-fold higher in microbial aggregates than in the surroundings inhabited by planktonic cells [50]. AHLs regulate the activity, growth rate, and EPS production of bacteria and the formation of microbial aggregates [51]. Once formed, AHLs can also regulate the resistance to environmental oscillations and other properties of aggregates [11,49].

In addition to interspecific communication at the bacterial level, microalgal-bacterial inter-kingdom signaling also occurs in microbial aggregates [48]. For instance, *Sulfitobacter* species in diatom-bacteria aggregates promote diatom cell division through secretion of the hormone indole-3-acetic acid, while the hormone synthesis is facilitated by diatom-secreted tryptophan which serves as its precursor [52]. Such communications between bacteria and primary producers shape the energy transfer and functions of the ecosystem in which the organisms reside. The interspecific communication mechanisms between the bacteria are not limited to chemical signaling, as some bacteria can respond to electrical signals [50]. For example, interspecific electron transfer in aggregates of *Geobacter sulfurreducens* and *Thiobacillus denitrificans* can be facilitated by electrically conductive magnetite nanoparticles, achieving acetate oxidation coupled to nitrate reduction [53]. In summary, various intercellular communication mechanisms in microbial aggregates promote, shape, and coordinate their formation and functionality.

### 3.4 Acclimatization and adaptation in aggregates

A diversity of microorganisms with multiple metabolic types sustains and protects the consortium overall [54], making the community in PBs more resistant to adverse conditions than a single species would be. For example, exposure to di-(2-ethylhexyl) phthalate (DEHP) was selected for the presence of *Acinetobacter* and *Bacillus*, which made the PBs more resistant to DEHP [55]. PBs have excellent potential in resisting the ecotoxicity of organic pollutants and nanoparticles, showing sustainable resistance to toxicity and strong plasticity in dealing with toxic interferences [56]. For example, PBs decolorize and convert crystal violet into nontoxic aliphatic compounds [57], and adapt to the chronic exposure of Fe_2_O_3_ nanoparticles via self-regulation of biofilm morphology, species composition and diversity [58]. In addition, the dense spatial construction of PBs acts as an intrinsic barrier to nanoparticle exposure [59].

The high microbial diversity and density of PBs give rise to complex cell-to-cell interactions, and the compact spatial distribution of cells in PBs promotes interspecific gene flow. This genetic and metabolic basis makes it possible to remold and adapt rapidly in response to environmental changes and stresses. One means of transformation and adaptation is horizontal gene transfer (HGT), the transfer of genetic material between microorganisms that are not in a reproductive relationship manner [60]. HGT is a mainspring for microbial evolution and niche adaptation and is thought to occur frequently in microbial aggregates [61]. The incorporation of diverse genes confers an adaptive benefit in response to survival stresses [48]. The three key mechanisms of HGT are conjugation, transformation and transduction, with plasmid conjugation being a common way of HGT in microbial aggregates [50]. HGT is well established in bacteria and widely recognized since its gene conjugation may help the recipient obtain resistance genes [60]. As for HGT in eukaryotes, there are ample foreign genes in eukaryotic genomic material, such as those for mitochondria and plastids, which are historic endosymbionts containing donated genomes [60]. HGT between organisms of different domains strengthens the acclimatization of PBs. The incorporation of bacterial genes into microalgal genomes is frequently observed in microbial aggregates, for example, when HGT from bacteria enabled the red alga *Galdieria sulphuraria* to adapt to extreme environments [14].

## 4 Regulation of the nitrogen cycle processes in PBs

**Fig. 3:**
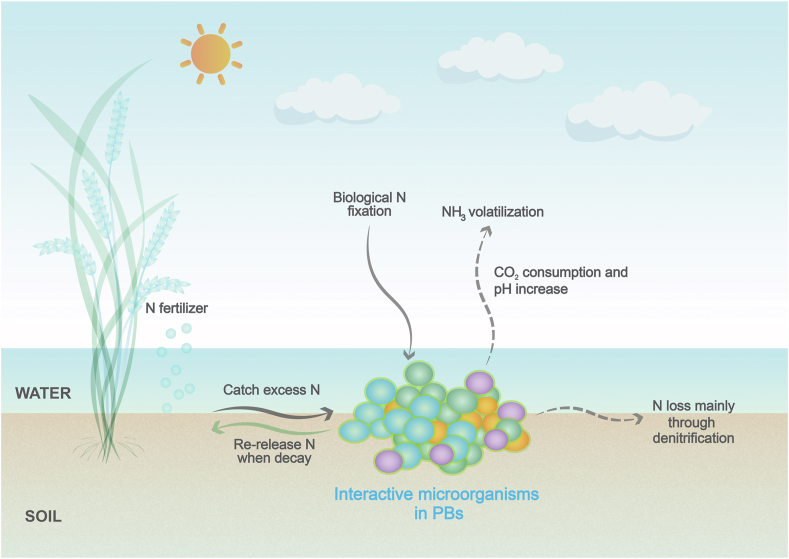
The process of the nitrogen cycle regulation by diverse microorganisms within PBs. Diverse microorganisms in PBs dominate the nitrogen cycle regulation through their interactive and integrated metabolism. By biological nitrogen fixation and assimilation of inorganic nitrogen, PBs catch and filter out excess nitrogen, keeping this nitrogen in reserve; the nitrogen is then released slowly for plant uptake and use. Ample organics provided by photosynthesis and an increased pH also foster partial nitrogen losses through denitrification and ammonia volatilization. Black solid lines indicate nitrogen sinks and black dashed lines indicate nitrogen losses.

Given that nitrogen is one of the limiting nutrients in rice yields and that ammonium fertilizers are being taken up and utilized by plants with low efficiency, it is vital to optimize the balance between the use of and pollution by nitrogen fertilizers in rice paddies. PBs act as a sink, yet are also a source of nitrogen in the paddy environment. Various transformation reactions take place simultaneously in PBs, which ultimately promotes nutrient cycling and improves nutrient utilization, reducing diffuse pollution [16] (Fig. 3).

Through biological nitrogen fixation and by assimilating inorganic nitrogen, PBs sequester and convert nitrogen into biomass and effectively keep *in situ* nitrogen in reserve [62]. Algae and autotrophic bacteria in PBs act as primary producers, a good portion of which are involved in nitrogen fixation, especially cyanobacteria and other photosynthetic bacteria [63]. Many species of cyanophytes are able to fix atmospheric N_2_, dominating the late growth and mature phases of PBs growth cycles in rice paddies when nitrogen is deficient in the overlying water [34], thus sustaining rice yields by supplementing and partially replacing chemical nitrogen fertilizers [64]. Nitrogen assimilation is the process where inorganic nitrogen is converted into organic forms by PBs and retained in the biomass, while the nitrogen present in the overlying water controls the growth of PBs [65]. Specifically, the reduced form of nitrogen (NH_4_^+^) can be assimilated by microalgae directly, while the oxidized forms of nitrogen (NO_3_^−^ and NO_2_^−^) are first reduced to ammonium and then incorporated into amino acids by using glutamate and ATP [42]. ATP and species diversity are central to nitrogen immobilization [62]. The decomposition of organics from photosynthesis will produce ample ATP. Thus, the high community diversity and photosynthetic activity of PBs galvanize the nitrogen cycle regulation. Due to man-induced drainage and drying in the later stages of the rice growing th season, PBs in rice paddies wither and decay gradually, causing nitrogen releasing back into the soil [16], and subsequently taken ly taken up by rice plants.

On the other hand, PBs may also cause nitrogen losses. The main mechanisms of nitrogen losses by PBs are ammonia volatilization and denitrification [66,67]. Aerobic conditions and sufficient organics from photosynthesis are the key drivers of nitrogen losses in PBs. Photosynthesis by algae, the dominant species in PBs, leads to carbon dioxide consumption and an increase in the pH of the overlying water by 0.18–0.25 units [68], which stimulates the volatilization of ammonium as ammonia [68,69]. Denitrification is a microbial process prevalent under hypoxic conditions, in which nitrate in rice paddies is reduced to N_2_ after previous oxidation of ammonium to nitrate via nitrification. Photosynthesis and respiration of PBs will affect the redox status at the soil-water interface [70], and ultimately enhance denitrification [67]. Specifically, oxygen released from photosynthesis facilitates the oxidation of ammonium and urea to nitrate, while the spatial distribution of the oxygen gradient results in the inner part of PBs being anoxic, facilitating the occurrence of denitrification [71]. Unlike other ecosystems such as sewage plants, where electron donors are limited or organics are consumed purely, the sustainable self-sufficient supply of organics in PBs overcomes electron donor limitation of denitrification [72], allowing nitrate to be continuously reduced to N_2_ by the denitrifying bacteria. It should be noted that although nitrification and subsequent denitrification by PBs can lead to nitrogen losses, they reduce pollution to some extent. Part of the surplus nitrogen is not lost to the aqueous environment, but rather converted into harmless N_2_.

Generally, the fixation and assimilation of nitrogen by PBs are of greater significance to the nitrogen cycle regulation in rice paddies than the relatively minor nitrogen losses via nitrification-denitrification. With the production of several hundred kilograms of biomass in one growing season, phototrophic biofilms immobilize dozens of kilograms of nitrogen per hectare [62], which is substantial and should not be ignored. The contribution of PBs to *in situ* nitrogen interception in rice paddies is also important, as they are able to reduce 48.47% ± 28.43% of nitrogen losses in overlying water [34]. Relative to a portion of nitrogen lost through ammonia volatilization and denitrification [64,67], more nitrogen, stored in the biofilms, may well be slowly released back into the system. Additional quantitative studies on the contribution of PBs to the biogeochemical cycle and utilization efficiency of nitrogen in rice paddies are definitely warranted.

## 5 Advantages of PBs in the nitrogen cycle regulation

**Fig. 4:**
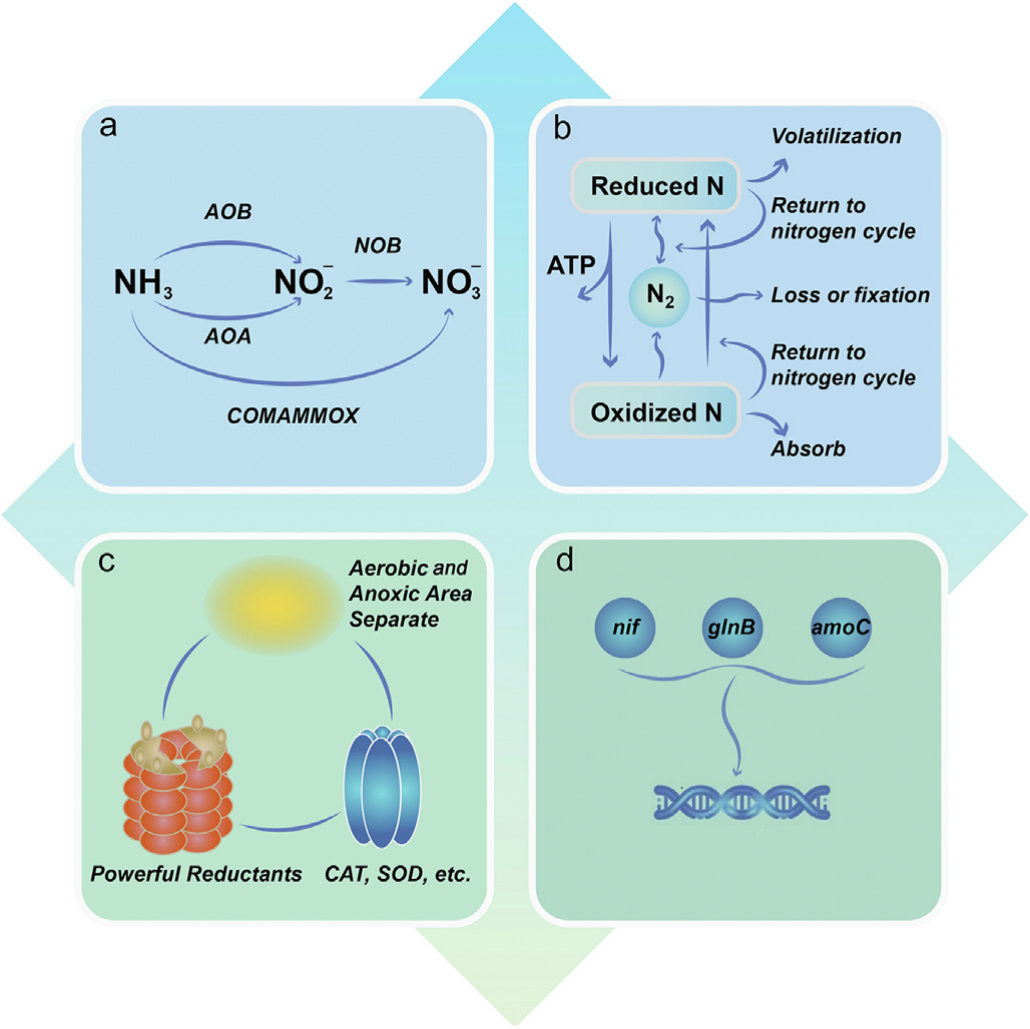
The flexibility and adaptation of the nitrogen cycle regulation by PBs. Benefiting from their three-dimensional structure, diversity of species and genes, ample self-produced organics and frequent interactions, PBs possess inherent qualities in the nitrogen cycle regulation, embodied in: (a) Adaptation to nutrient and environmental changes through different ammonia oxidation pathways. (b) Linked nitrogen cycle processes. Microorganisms in aggregates cooperate to survive, and the boundary between products and substrates is blurry. (c) Adaptation to oxygen threat through the differential distribution of oxygen, reductants such as ferredoxin, and detoxification via catalase (CAT), superoxide dismutase (SOD) and other enzymes. (d) Strengthening the regulation of the nitrogen cycle by HTG and expressing auxiliary metabolic genes. AOA, ammonia-oxidizing archaea; AOB, ammonia-oxidizing bacteria; NOB, nitrite-oxidizing bacteria; ATP, adenosine triphosphate; *nif*, nitrogenase gene; *glnB*, glutamine synthetase gene isoform B; *amoC*, ammonia monooxygenase gene subunit C.

PBs possess inherent levers for nitrogen cycle regulation. Various microorganisms within PBs cooperate to perform a number of complex nitrogen cycle reactions, and sufficient self-produced organics provide necessary energy and electrons in support of a sustainable nitrogen cycle. The metabolic and genomic flexibility and adaptation of PBs reinforce their success in the nitrogen cycle regulation (Fig. 4).

### 5.1 Hotspot for rapid transformation of microbial nitrogen

Much attention has been drawn to deficiencies of pure cultures of individual microorganisms for the nitrogen cycle processes. Since the ammonia-oxidizing bacteria (AOB) and nitrite-oxidizing bacteria (NOB) responsible for the two steps of nitrification were isolated in the late 1800s [73], there has been tremendous progress in our understanding of the various steps of the nitrogen cycle and the organisms involved, many of which have been characterized with respect to their functional genes and corresponding enzymes. In addition to the discovery of ammonia-oxidizing archaea (AOA) [74], recent examples in this long line of “novelties” are complete nitrification by a single microorganism [75,76] and nitrification without oxygen [77]. Research on pure cultures of individual microorganisms involved in the nitrogen cycle has been and is rewarding to elucidate their distinct ecophysiological requirements [21]. However, due to the specifics of the environment, nitrogen turnover by individual microorganisms is bound to involve only a part of the nitrogen cycle reactions. Individual microorganisms will never perform all reactions required for a complete nitrogen cycle. Indeed, nitrogen transformation in rice paddies and other natural environments is carried out via the division of labor in microbial aggregates, with communities being more comprehensive than individual microorganisms regarding the nitrogen cycle.

The material basis of PBs boosts the nitrogen cycle regulation. To perform a complete nitrogen cycle, an all-inclusive set of microorganisms providing all the necessary enzymes, plus the conditions allowing these enzymes to be meaningfully expressed, is needed. High biodiversity is a prerequisite for a complete nitrogen cycle in PBs. The main processes of the nitrogen cycle can all be found in PBs: nitrogen fixation, nitrogen assimilation, ammonification, nitrification, denitrification, and anaerobic ammonium oxidation. In addition, the material basis of PBs provides the basis for the nitrogen cycle regulation. That is, adequate self-produced organics provide ATP and act as electron donors for the nitrogen cycle processes. Overall, the nitrogen cycle is driven by the availability of electron donors, electron acceptors and ATP, which is amply provided for in PBs. Sixteen molecules of ATP are needed per molecule of N_2_ fixed in nitrogen fixation. This may be the reason why many nitrogen-fixing bacteria live in symbiosis with eukaryotes in PBs. Photosynthesis and the accumulation of biomass provide sufficient organics, which serve as the chief sources of ATP [78]. The organics are oxidized to release chemical energy associated with electron transfer. While the organics act as designated electron donors, nitrate is in competition with O_2_ as an electron acceptor [79]. Electron donor availability effectively limits nitrate reduction. In each step of the nitrate reduction pathway via nitrite, nitric oxide and nitrous oxide to dinitrogen gas and even ammonium, electron donors are needed and precious. Additionally, nitrite from nitrate reduction is a major source for anaerobic ammonia oxidation (Anammox) and other nitrogen cycle processes [80], so the material basis of PBs drives and regulates their intrinsic nitrogen cycle.

### 5.2 Adaptability and metabolic flexibility of multi-species aggregates

The broad environmental adaptability of PBs is supported by microbial diversity. Given the continuous fluctuations in nitrogen concentrations and environmental factors in rice paddies, different types of nitrogen-cycle microorganisms in PBs show different adaptive advantages. For example, in ammonia oxidation and under aerobic conditions, ammonia is oxidized to nitrite and nitrate by AOB and NOB respectively [81]. However, AOB may be less effective at low levels of ammonia and oxygen, conditions at which AOA and complete ammonia-oxidizing (comammox) bacteria will take over. Due to their high tolerance to ammonium limitation [82,83] and higher substrate affinity [84,85], AOA are believed to play a leading role in the nitrification process in oligotrophic environments [86] and in low pH environments [87]. Besides, due to their all-inclusive metabolic labor and higher growth yields [88], comammox bacteria could have a competitive advantage in microbial aggregates with low substrate concentrations [75,76]. Actually, such niche differentiation plays a significant role in species coexistence and microbial diversity [89,90], supporting the broad adaptability of PBs.

Nitrogen cycle stability is maintained by interspecific collaboration. A complex food web and syntrophic interactions sustain the various steps needed for nitrogen cycle regulation. In addition to the symbiotic relationships between microalgae and bacteria [91], essential cooperation between nitrogen-cycle bacteria for nitrogen transformation processes is prevalent. The steps in the nitrogen cycle are linked: the products of one step are the substrates for the next step in the cycle. A shortage of integral functional microbial communities or a deficiency of symbiotic nitrogen nutrient relationships will lead to an interruption of the nitrogen cycle. Bioavailable forms of nitrogen, such as ammonium and nitrate, originate primarily from fertilization, ammonification, and nitrification. AOB, in tandem with NOB, convert ammonium-based fertilizers to nitrate [21], which is one of the dominant forms of bioavailable nitrogen, supporting the growth of eukaryotes, bacteria, and archaea possessing assimilatory nitrate reductases [92,93]. Meanwhile, the energy produced in autotrophic nitrite oxidation contributes to carbon fixation [94], reinforcing connections with the carbon cycle. Similarly, dissimilatory nitrate reduction to ammonium (DNRA) is an integral step in the nitrogen cycle in PBs and links *in situ* oxidation and reduction processes [95], further boosting the nitrogen cycle. These are the types of interlinkages contributing to the stability of the nitrogen cycle in PBs.

Oxygen tolerance is conferred by three distinct mechanisms. Oxygen from photosynthesis and the atmosphere is indispensable for metabolic processes such as aerobic ammonia oxidation [96] and aerobic methane oxidation [97], but it is also a distinct stressor as most nitrogen cycle steps favor or even require hypoxia [98]. PBs adapt to oxygen through three distinct mechanisms. Firstly, the three-dimensional structure of PBs results in oxygen gradients. Anammox is inhibited by oxygen, but anammox bacteria reside in the anoxic interior of biofilms whereas AOB reside in the aerobic surface layer [99,100], resulting in a delicate balance between aerobic ammonia oxidation at the outer layer and anaerobic ammonia oxidation at the inside of the biofilms [101]. Secondly, the management of intracellular oxygen which carries a substantial energetic cost and requires maintaining a delicate intracellular redox state. Taking the nitrogen fixation process as an example, nitrogenases are inactivated by oxygen exposure. In addition to separating nitrogen fixation from photosynthesis spatially and temporally [102,103], powerful energy-consuming reductants such as ferredoxin, and protection mechanisms such as detoxification via superoxide dismutase [104] protect nitrogenase against the adverse effects of oxygen. Finally, alternative aerobic steps in the nitrogen cycle like aerobic denitrification, could and most likely do expand the acclimatization and adaptation of PBs to oxygen. Although its molecular mechanism is not yet fully understood, aerobic denitrification does exist in biofilms [105], and its biological energy production and reaction kinetics have been illustrated [106]. Moreover, basal transcription of denitrification genes under aerobic conditions has been observed [107]. Provided that it is expressed by periplasmic nitrate reductase (Nap) rather than membrane-bound nitrate reductase (Nar) in the electron transfer pathway to capture the electrons, aerobic denitrification may occur [108].

In the context of fluctuating nitrogen concentrations, PBs adopt functional trait allocation and ecological strategies related to their genomic underpinnings to achieve a modicum of stability [109]. For example, in contrast to most cyanobacteria, *Microcoleus chthonoplastes* typically lacks the canonical *nif* genes to fix dinitrogen, but the organisms can and do acquire the *nif* operon through HGT and then express it under natural conditions if and when necessary [110]. Besides, viruses may reprogram the microbial metabolism of their microbial hosts by gene transfer and expression of auxiliary metabolic genes (AMGs) [111], thus modulating the nitrogen cycle genes such as *glnB* [112] and *amoC* [113]. In addition to fluctuating nitrogen concentrations, other variable environmental conditions similarly force PBs to actively adapt in real time. For instance, PBs are adept at using genomic and metabolic adaptation strategies to adapt to adverse environmental conditions such as low temperatures [114]. Where DNRA supplants denitrification, nitrogen recycling is sustained and nitrogen losses are reduced within the biofilm through ammonia assimilation [115]. The expression of carbon fixation genes and succedent biomass accrual [116] in the algal-bacterial consortia are conducive to efficient recycling of nutrients [115], which ensures essential organics and substrates for such energy-consuming adaptations. As a result, PBs are able to deal with and counteract fluctuations in their habitat effectively, which highlights their strategies to optimize the nitrogen cycle regulation [117].

## 6 Challenges and future prospects

### 6.1 Molecular mechanisms underpinning the nitrogen cycle

The occurrence in PBs of all the main reactions that together make up the nitrogen cycle is essentially clear. However, new microbial pathways of nitrogen transformation may well be waiting to be discovered, and this may also affect our understanding of the nitrogen cycle in biofilms. For example, different from previous views that AOA oxidize hydroxylamine to nitrite using hydroxylamine oxidoreductase, the product could be nitric oxide, and the enzyme catalyzing the oxidation of nitric oxide to nitrite is still unknown [118]. With the environmental complexity of rice paddies, intricate species diversity of PBs and the inherent variability of nitrogen valence in these systems may be the cradle of undiscovered nitrogen transformation processes, and the exchange of genetic information among species and redundancy of functional genes provide the possibility of the occurrence of uncovered processes. A hypothetical potential example could be the enhanced interspecific gene transfer by viruses impacting the nitrogen cycle; however, such an impact of viruses in the nitrogen cycle regulation has rarely been studied. To identify new processes, thermodynamic considerations and new approaches have shown their value. Subsequent to the conjecture that anammox might exist, based on thermodynamic data showing that anammox is an energy-yielding reaction [119], its existence has been confirmed [120]. In addition, following the theoretical prediction based on kinetic and thermodynamical theory [88], comammox was eventually discovered with the help of advanced metagenomic techniques [75,76]. Similarly, reaction (1) is an exergonic reaction for vital movement based on thermodynamic data, with an energy yield per mole of N (ammonium, nitrite and nitrate) converted slightly higher than that of reaction (2) (which represents anammox), suggesting that there should be a definite niche for this reaction. The theoretical chemotrophs capable of reaction (1) may exist in an environment with sufficient ammonium, alkaline and limited denitrifying bacteria, competing with the nitrifiers and anammox bacteria in PBs, especially in the anoxic or anaerobic interior.

(1) $${5NH}_{4}^{+}+{3NO}_{3}^{-}\to {4N}_{2}+{9H}_{2}O+{2H}^{+};{\Delta G}^{0^{\prime }}=-1485\;kJ/reaction$$

(2) $${NH}_{4}^{+}+{NO}_{2}^{-}\to N_{2}+{2H}_{2}O;{\Delta G}^{0^{\prime }}=-358\;kJ/reaction$$

### 6.2 Inter-specific interactions in PBs

The complex composition of PBs underlies the complexity of their functions but poses great challenges to the analysis of the mechanisms underpinning these functions. The precise effects of the various components have not been clearly elucidated. For instance, the roles of specific species, their relationships and interaction mechanisms with other organisms, and the extent to which their individual and collective ecophysiologies are of critical significance to the vitality of rice paddies are still in the dark. Moreover, clear unknowns exist with regard to *in situ* mechanisms, such as the coordination between biology and environment, the interaction mechanisms between producers, consumers, and decomposers, and extracellular and intracellular coordination mechanisms. Further exploration could greatly benefit from the development of new technologies and methods.

One thing is clear though, the key to clarifying those mechanisms is to cultivate the various organisms in the laboratory, where many of these microorganisms currently have not been grown successfully. Such is a thorny issue that urgently needs to be resolved. That being said, the nitrogen cycle regulation by PBs is not performed by single microorganisms performing their functions in isolation, but rather the result of symbiotic cooperation of and in microbial aggregates. The metabolism of the microorganisms involved is very flexible, and there is no clear, fixed division of metabolic labor. For example, various NOB convert urea to ammonia and CO_2_ to support AOA and receive nitrite in return [121]. Such strategic metabolic versatility may well be more common and deserves more attention.

### 6.3 Quantitative contributions to agriculture and environment

The contribution of PBs as nitrogen-fixers and nitrogen-removers in rice paddies can be quantitatively evaluated through large-scale *in situ* monitoring, meticulous laboratory culture analysis and the establishment of appropriate models, so as to determine the agricultural and ecological significance of PBs. However, it is and will be challenging to disentangle and regulate the composition, structure and growth of PBs in rice paddies. Subsequent to and based on a thorough understanding of composition, growth habits and influencing factors, it will be necessary to focus on the growth regulation of PBs in rice paddies spatially and temporally, so as to make better use of their biology for the nitrogen cycle regulation. The rapid progress of molecular biology approaches should facilitate this line of research.

## 7 Conclusions

As microbial aggregates in rice paddies, the distinguishing and defining feature of PBs rests in their complex composition and completely integrated food web and energy flux. The result of this integration is that they transform if and when necessary, adapting to variations in the environment. Accordingly, PBs sustain a wide variety of nitrogen cycle steps simultaneously but to different degrees in time and space, capturing surplus nitrogen at the earlier stages of their life cycle and subsequently releasing this nitrogen at the later stages. As a result, PBs play consequential roles in reducing nitrogen pollution and improving nitrogen utilization in rice paddies, and offer promising ways to maintain the safety of farmland ecosystems and to sustain and possibly increase agricultural production. Ongoing research should focus on unknown pathways, clearer mechanisms and quantification of the contribution of PBs to *in situ* nitrogen cycle regulation.

## Declaration of competing interest

The authors declare that they have no known competing financial interests or personal relationships that could have appeared to influence the work reported in this article.
